# Eco-sustainable chromatographic method for the determination of favipiravir and nitazoxanide for COVID-19: application to human plasma

**DOI:** 10.1186/s13065-024-01364-3

**Published:** 2025-01-09

**Authors:** Amal B. Ahmed, Maha M. Abdelrahman, Fadwa H. Edrees

**Affiliations:** 1 Pharmaceutical Chemistry Department, Faculty of Pharmacy, Nahda University (NUB), Sharq El-Nile, Beni-Suef, 62511 Egypt; 2 Pharmaceutical Analytical Chemistry Department, Faculty of Pharmacy, Beni-Suef University, Alshaheed Shehata Ahmad Hegazy St, Beni-Suef, 62514 Egypt; 3 Pharmaceutical Chemistry Department, Faculty of Pharmacy, Nile Valley University (NVU), El Fayoum, 63518 Egypt

**Keywords:** COVID-19, Favipiravir, Nitazoxanide, Plasma, Therapeutic drug monitoring, Green HPLC method

## Abstract

Coronavirus disease 2019 (COVID-19), an extremely contagious illness, has posed enormous challenges to healthcare systems around the world. Although the evidence on COVID-19 management is growing, antiviral medication is still the first line of treatment. Therefore, it is critical that effective, safe, and tolerable antivirals be available to treat early COVID-19 and stop its progression. Recently, favipiravir (FAV) has received FDA approval as safe and effective antiviral medication for COVID-19 management. Nitazoxanide (NTZ) also possesses antiviral and immunomodulating activities. Moreover, FAV and NTZ in combination are clinically used in COVID-19 treatment with reported safety, synergistic antiviral and immunomodulating effects. Despite the availability of various clinical studies on both FAV and NTZ, no existing analytical application for the simultaneous estimation of FAV and NTZ exists. As a result, the current work goal is to establish a green HPLC method for their analysis and implementation to human plasma. The developed method utilizes isocratic elution with 0.1% aqueous formic acid: ethanol (55:45, v/v) and dantrolene as internal standard. The bioanalytical validation parameters passed the FDA acceptance criteria. NEMI, eco scale, AGREE and ComplexGAPI approaches were used for qualitative and quantitative evaluation of the method’s greenness.

**Supplementary Information:**

The online version contains supplementary material available at 10.1186/s13065-024-01364-3.

## Introduction

Coronavirus disease 2019 (COVID-19) represents a recent global epidemic caused by severe acute respiratory coronavirus 2 (SARS-CoV-2) [1]. In severe cases, the virus can cause pneumonia, serious lung damage, respiratory distress, and cytokine storm [2]. Antiviral COVID-19 medications have been shown to effectively inhibit SARS-CoV-2 growth and halt disease progression [3]. However, antiviral treatment efficacy and timing are critical in this regard. Oral antiviral agents allow SARS-CoV-2 infection to be managed without hospitalization, reducing the overall burden that COVID-19 can place on the medical field [4–7]

**Fig. 1:**
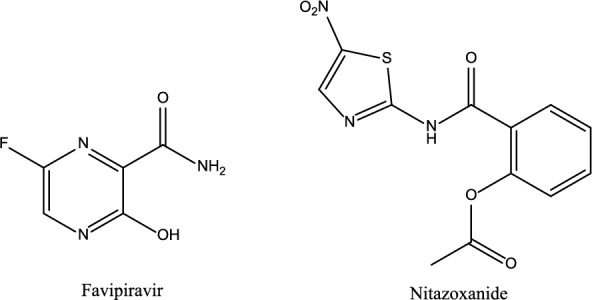
Chemical structures of FAV and NTZ

Favipiravir (FAV), (6-Fluoro-3-hydroxypyrazine-2-carboxamide) is a purine derivative [8], Fig. 1. It has a slight water solubility (log P 0.72) and weak acidic properties (pka 5.1). It has a broad-spectrum inhibition on RNA-dependent RNA polymerases. This has recently received US FDA-approval as a safe and effective treatment of COVID-19 [9]. The use of FAV speeds up the viral clearance and clinical improvement time [10]. Additionally, it significantly decreases the observed damage on radiology images after recovery [11]. Moreover, adding FAV to the treatment protocol improves outcomes for COVID-19 inpatients. On the other hand, FAV is specifically consumed in RNA virus-infected cells with elevated RNA production, which confirmed its specificity [9]. FAV is less likely to generate FAV-resistant strains, which represents its primary advantage as an antiviral agent [12]. However, some well tolerated side effects have been reported in COVID-19 patients treated with FAV, including nausea, vomiting, and increased alanine and aspartate aminotransferase levels. In addition, FAV may cause hyperuricemia, which may lead to acute gouty arthritis in some patients [11].

Drug repurposing is the process or the way for identifying new indications for approved drugs. It is a very effective drug discovery strategy because it takes less time and money to find a therapeutic agent than novel drug discovery process [13]. Various unexpected old drugs are being reused in the COVID-19 era to find new choices with efficient and inexpensive properties against SARS-CoV-2 [14, 15]. Nitazoxanide (NTZ) is among these drugs, which is a FDA approved anti-parasitic drug. NTZ is a nitrothiazole benzamide derivative (2-acetyloxy-N-5-nitro-2-thiazolyl) (Fig. 1). It has low water solubility (log P 1.63) and basic properties (pka 10.62). NTZ exhibits reported broad-spectrum antiviral activity against various DNA and RNA viruses [16, 17]. In cell culture assays, it demonstrated good in vitro activity against SARS-CoV-2, implying potential for repurposing in COVID-19 [18]. Furthermore, NTZ has the potential to stimulate the host's innate immune responses, thereby combating the possible fatal cytokine storm. Other significant aspects of the drug include the potential for improving lung and multiple organ damage, providing an additional benefit to COVID-19 patients with comorbidities [17, 19]. NTZ activates endogenous IFN synthesis and release to stop SARS-CoV-2-induced IFN decrease. The IFN pathway is essential for the prevention of various viral infections, particularly SARS-CoV [16]. On the other hand, NTZ is safe for human and can be tolerated in doses up to 4 g with minimal gastrointestinal side effects [20]. Besides, NTZ demonstrates an overall favorable safety profile, and affordable price [21]. In addition, no significant side effects were reported for NTZ, and they are primarily associated with the gastrointestinal tract, such as stomachache, diarrhea, nausea, flatulence, and increased appetite. NTZ may cause enlarged salivary glands, fever, elevated creatinine levels, elevated levels of alanine aminotransferase in serum, pruritus, rhinitis, dizziness, and discolored urine [22].

Several trials have tested NTZ combined with other antiviral agents, yielding promising results in COVID-19 [23, 24]. In addition to its non-specific positive effects on host immunity, NTZ functions as an antiviral drug through a distinct process that commences subsequent to the blocking of viral nucleocapsid N protein production by FAV [17, 25]. Therefore, FAV and NTZ are expected to act at least [26] cumulatively, and possibly synergistically when given together [25]. This combination demonstrated its important antiviral and immunomodulating effects without significant toxicity [27].

Lately, environmental consciousness has been growing and moved up the chemical society's list of priorities. Green chemistry was developed to decrease or remove the use and production of hazardous compounds, chemicals and the procedures must be designed with the environment in mind at every stage of the entire analytical process [28] taking early sampling and sample-preparation measures into account. Several metrics have now been optimized in accordance with the ecological impact evaluation of an analytical process. One of these metrics is the National Environmental Method Index (NEMI), an old qualitative technique established to provide a clear representation of the greenness of the analytical method [29]. The analytical eco scale is another approach that depends on penalty points subtracted from a total of 100. The higher the score, the greener and more environmentally friendly the analytical procedure [30]. An innovative software was developed as an analytical greenness calculator. The AGREE is a versatile and simple assessment tool that yields an easily interpretable and informative result [31]. In 2021, ComplexGAPI was lunched as a new software that was developed from the Green Analytical Procedure Index (GAPI) to provide greater legibility, simplicity, and user-friendliness. Additionally, greenness evaluation of the preanalytical procedures is another objective. It included extra fields related to the processes carried out before the analytical procedure itself [30, 32].

COVID-19 is a relatively new disease with diverse medical studies. In addition, several research have been conducted on the drugs used in COVID-19 treatment. However, a review of the literature showed that there is no reported analytical method for simultaneously determining FAV and NTZ for COVID-19 management. There are some methods for estimation of each of them separately or with other drugs including TLC densitometric [33–35], HPLC [36–39], electrochemical [40–42] and spectrophotometric [38, 43, 44] methods for FAV. Similarly, NTZ was determined by several chromatographic methods such as TLC densitometric [45–48], spectrophotometric [46, 49–51], electrochemical [52, 53], spectrofluorimetric [54, 55], HPLC [54, 56, 57] and LC–MS [58, 59] methods.

FAV serum concentration shows significant variability and decreases in patients with mild to moderate COVID-19 disease. Besides, its pharmacokinetics may be affected by gender [60]. On the other hand, recent observations indicated a hyperdynamic and hyper-inflammatory states in COVID-19 resulted in rapid elimination of renally cleared drugs [61, 62]. So, therapeutic drug monitoring (TDM) of renally cleared drugs may be advised in severe COVID-19 cases. Hence, it was recommended to develop a sensitive, selective, and cost-effective analytical method for estimating any reported safe and effective drug combinations for management of this disease. In addition, development of these methods for the drug determination in biological samples is also beneficial in bioequivalence studies and in the development of new drugs [63]. However, no method is available for simultaneous determination of FAV and NTZ. Herein, we aimed in this work to develop a novel green HPLC method for simultaneous quantification of FAV and NTZ in plasma for the regular drug quality control and further clinical trial monitoring.

## Materials and methods

### Instruments

The precipitated plasma proteins were separated using 80-2C low-speed electric centrifuge, 4000 rpm (Zjmzym, China). 250 VM centrifugal mixers (Hwashin, Seoul, Korea) and Rongtai adjustable capacity micropipette devices (0.1–100 μl volume, Shanghai, China) were employed.

Thermo Scientific Dionex Ultimate S 3000 HPLC, equipped with Ultimate Quaternary solvent delivery pump, ACC-3000 autosampler and DAD −3000 diode array detector (Germany) was used. The stationary phases tested were Hypersil Gold^®^ C18, (2.1 × 50 mm, 1.9 μm), Hypersil Gold^®^ C18 (4.6 × 150 mm, 3 μm), XBridge^®^ HPLC RP- C18 (4.6 × 250 mm, 5 μm), Xterra^®^ CN (4.6 × 150 mm, 3 μm) and Xterra^®^ RP- C8 (2.1 × 50 mm, 1.9 μm). The output signal was monitored and processed using Chromeleon chromatography data system software. Additionally, an electronic balance from Sartorius, Germany, a Sonix TVSS-series ultrasonicator from California, USA, and Jenway 3505 pH metres from Staffordshire, UK were incorporated.

### Materials and reagents

Favipiravir pure sample was received as a gift from EIPICO Pharmaceuticals (Tenth of Ramadan City, Egypt), and NTZ was given by Copad Pharma for Pharmaceutical Industries (El Obour City, Egypt). Dantrolene (DNL) was the internal standard used, it was kindly provided from Hikima Pharmaceuticals (Giza, Egypt). FAV, NTZ and DNL purity grades are 100.5%, 99.9% and 99.7%, respectively. Ethanol and acetonitrile were bought from Sigma Aldrich and were of high purity for HPLC (Fisher, Loughborough, UK). Orthophosphoric acid, formic acid, dihydrogen potassium phosphate, potassium hydroxide, sodium lauryl sulphate, ammonia solution (33%) and acetic acid were of optimum analytical quality and bought from EL-Nasr Pharmaceutical, Chemical Co., Abu Zabaal, Cairo, Egypt.

### Blank human plasma samples

Six healthy individuals' human blank plasma samples were collected by Sama Laboratory in Elfayoum, Egypt.

### Chromatographic conditions

Thermo Scientific Dionex Ultimate 3000 UHPLC (Germany) instrument was used with XTerra R^®^ HPLC C18 column (4.6 × 250 mm, 5 μm). The injection volume was 20 μL and the separation was done by an isocratic elution program 0.1% aqueous formic acid: ethanol (55:45, v/v). The total run time was 8.5 min. The UV detection was performed at 230 nm, and the column temperature was adjusted to 30 ºC.

### Standard solutions

Three distinct 25 mL calibrated flasks were used to generate stock standard solutions of FAV, NTZ, and DNL (1 mg mL^−1^) in methanol. For additional applications with the established approach, their working standard solutions (0.1 mg mL^−1^) were generated in the mobile phase combination. These standard solutions were freshly prepared before each analysis.

#### Calibration standards

In a pair of separate groups of 5 mL volumetric flasks, various concentrations of 4–60 μg mL^−1^ and 2–60 μg mL^−1^ oF FAV and NTZ, respectively were added separately using their corresponding standard working solutions (0.1 mg mL^−1^). Each flask received 0.5 mL of blank plasma, 25 μL of IS (DNL) from its standard stock solution (1 mg mL^−1^), and methanol was added to complete the volume to the mark. Prior to being centrifuged for 20 min at 4000 rpm, samples were vortexed for 1 min. To obtain the calibration standards 4, 6, 10, 20, 30, 40, 50, 60 μg mL^−1^ for FAV and 2, 4, 6, 10, 20, 30, 40, 50, 60 μg mL^−1^ for NTZ a triple of each processed sample was injected after filtering through a 0.45 m millipore filter. Then, the steps of the HPLC method were conducted accordingly. After calculating the drug's peak area to internal standard's peak area, the regression equations were built by constructing the obtained peak areas ratio to the relative drugs' concentrations.

#### Lower limit of quantification (LLOQ)

Each drug response at LLOQ should not be less than five-times that of blank plasma (attained by plasma of healthy donors that did not administer any medications for the last 2 weeks); the peak should be discernible; and the estimated concentrations should have a precision of a maximum of 20% of RSD or the coefficient of variation.

#### The upper limit of quantification (ULOQ)

The ULOQ peak should be consistent, with an accuracy of within 100 ± 15% of actual concentration and the reliability of the coefficient of variance or 15% RSD.

#### Quality control samples

The chosen quality control samples were 8 (LQC), 25 (MQC) and 50 (HQC) μg mL^−1^. All the prepared solutions and samples of quality control (QCS) were instantly maintained at −20 °C.

#### Specificity and selectivity

By comparing the HPLC chromatograms of the blank plasma samples (from 6 healthy donors) with the plasma samples boosted with each of the examined medicines at the appropriate LLOQ and IS, the specificity was evaluated.

#### Accuracy and precision

Within-run accuracy and precision were tested three times on the same day by analyzing the prepared QCs. In contrast, the difference between run precision and accuracy was tested by analyzing the same samples over a 3-day period (between-run accuracy and precision). When the calculated %RSD or CV of the tested concentrations was less than 15%, the methods were considered precise, and accuracy was deemed to be satisfactory when the bias value of the evaluated concentrations was less than 15%.

#### Extraction recovery

To ensure that the plasma matrix had no effect on the recovered compounds, the extraction recovery was studied. Peak area ratios from samples of plasma that had been spiked were contrasted with those from pure standards at equivalent amounts. LQC, MQC, and HQC concentrations were selected.

#### Stability

Three sets of samples of different QC level (HQC, MQC, and LQC samples) were analyzed after being exposed to a variety of stability circumstances, including ambient temperature storing (25 °C) for 6 h (Bench-Top stability), rendering at the temperature of the auto-sampler for 1 day (Postpreparative stability), and three rounds of freezing at −20 °C followed by thawing for twelve hours to environment's temperature (Freeze and Thaw Stability). The analytes were considered stable in plasma when the concentrations obtained were within 100 ± 15 of their assumed values, with 100% on average recoveries.

## Results and discussion

The COVID-19 plague persists as an imminent threat for society, claiming 4 million lives worldwide. Despite widespread vaccination, it is predicted to remain a problem due to gaps in resource allocation and the possibility of new mutants evading vaccine-mediated protection. Consequently, COVID-19 treatment and prevention strategies have been extensively researched. Several studies have found that effective COVID-19 treatments should include both antivirals and immunomodulators [64–66]. Thus, NTZ is combined with FAV to achieve both antiviral and immunomodulatory effects [27]. Clinical investigations reported that this combination has high efficacy without toxicity. Despite the abundance of clinical studies investigating both FAV and NTZ, no analytical method exists for their concurrent quantification. This study involves developing a green, sensitive, and selective HPLC approach to simultaneously quantify each of them in human plasma.

### Methods development and optimization

Optimization of the proposed method was done to attain the best resolution between the analyzed drugs in the shortest analysis time. It was crucial to assess how various parameters affected the degree of selectivity, sensitivity, and effectiveness of the separation.

#### Stationary phase

Various stationary phases (listed under Instrument) of various column particle sizes and lengths were tested. Among them, XBridge^®^ HPLC C18 (4.6 × 250 mm, 5 μm) column provided a superior resolution considering all the analytes.

#### Mobile phase

Green chemistry concepts and practices have recently emerged, capturing the attention of researchers across many disciplines. Green chemistry is to design chemical techniques and products that decrease or prevent using of hazardous materials and their formation. Therefore, the authors intended to apply the green analytical chemistry concepts during the development of this work. Consequently, green solvents were assessed to establish optimal separation between the studied analytes with symmetric peaks. Water and ethanol (the ideal green solvents) were evaluated in different ratios with promising results. The most effective combination for separation of FAV and NTZ was found to be water to ethanol in a proportion of (55:45, v/v). But FAV peak overlapped with the peak of human plasma, so using a pH modifier was necessary. Different ranges of acidic and basic pH values were tested using different reagents in concentrations of (0.05%, 0.10%, 0.15%) for each of orthophosphoric acid, acetic acid, formic acid, and triethylamine. Additionally, sodium lauryl sulphate was tried to achieve better resolution and peaks symmetry. Formic acid was found to achieve the best separation between peaks of FAV and plasma. Therefore, different concentrations of aqueous formic acid were tried (0.05%, 0.10%, 0.15%). Finally, the optimum resolution between the tested components with acceptable peak symmetry was achieved using 0.1% aqueous formic acid and ethanol in the ratio of (55:45 v/v) at flow rate of 0.8 mL min^−1^.

#### Column temperature

The column temperature was optimized where 25, 30 and 35 °C were tried. It was found that elevation of column temperature to 30 ^0^C accompanied with decrease in peaks tailing. However, further elevation above 30 ^0^C did not produce any improvement of the peaks shape, so 30 °C was the chosen temperature.

#### Scanning wavelength

Various scanning wavelengths were verified (220, 230 and 330 nm) to select the most sensitive one. UV detection at 230 nm achieved the best sensitivity for the tested drugs without interference from the plasma peak.

**Fig. 2:**
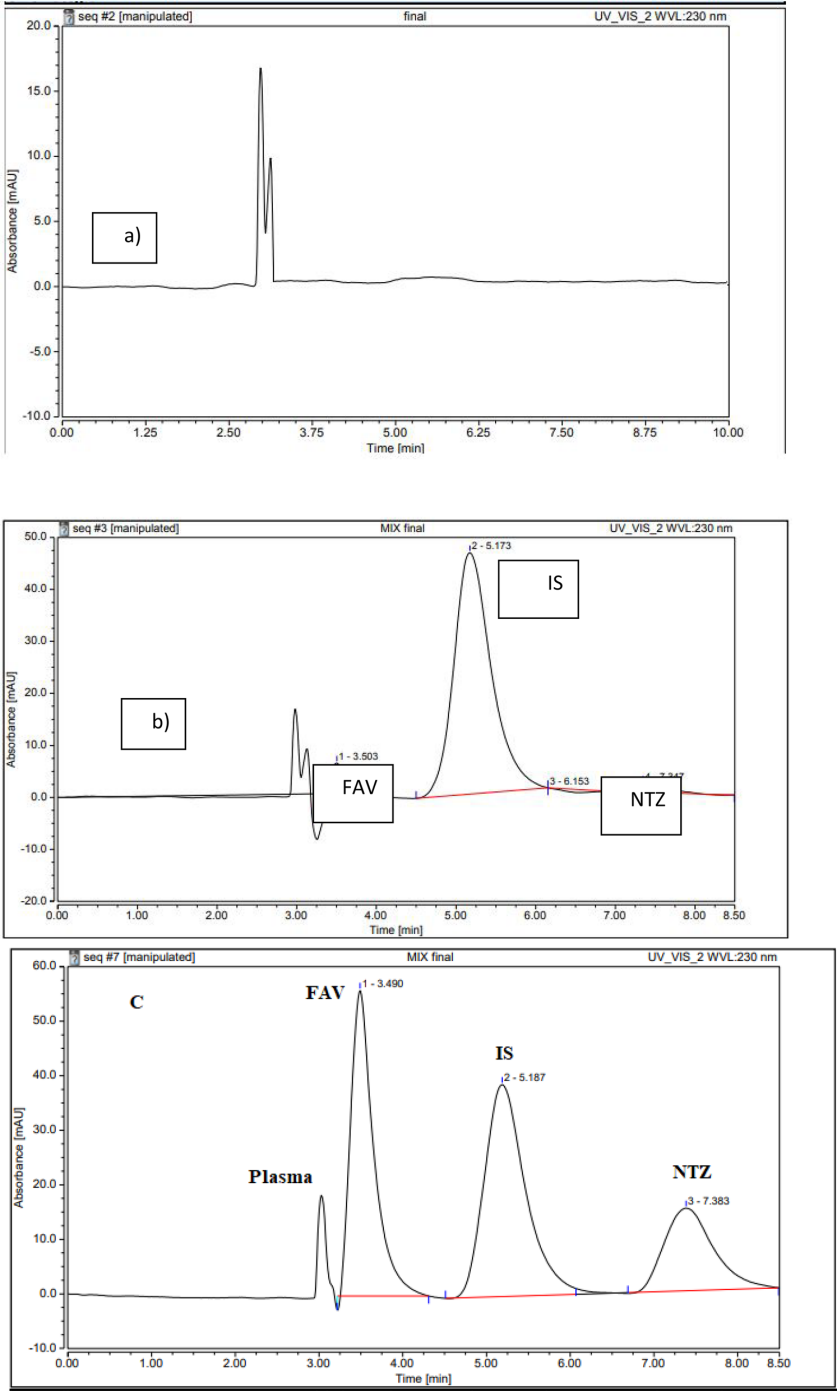
HPLC-chromatograms at 230 nm of **a** blank plasma, **b** human blank plasma spiked with standard solution of FAV, NTZ at LLOQ and IS. and **c** human blank plasma spiked with standard solution of FAV, NTZ at LLOQ and IS

Finally, satisfactory peak shapes resolution was obtained where the t_R_ of FAV = 3.50, DNL (IS) = 5.17 and NTZ = 7.35, as revealed in Fig. 2.

### Method validation

The sensitivity, specificity, linearity, accuracy, precision, and matrix effect of the method were all investigated. It was conducted in accordance with the United States Food and Drug Administration's Guidance for Industry-Bioanalytical Method Validation [67].

#### Linearity of calibration curves

The newly developed method's standard calibration curves for boosted human plasma were linear across the concentration levels of 4–60 µg mL^−1^ for FAV and in the range of 2–60 µg mL^−1^ for NTZ. The least-squares regression results yielded the following regression equations:

$${\text{for FAV}},\,{\text{Y}}\, = \,0.{4585}\,{\text{X}}\, + \,0.{2245}\quad {\text{r}}\, = \,0.{9998}$$

$${\text{for NTZ}},\,{\text{Y}}\, = \,{1}.{2735}\,{\text{X}}\, + \,0.0{844}\quad {\text{r}}\, = \,0.{9996}$$

The results confirmed the linearity of the obtained calibration curves, accurate and precise over these ranges. For all calculated standard concentrations, the acceptance criteria were a 15% deviation from the theoretical concentration (100 ± 15%).

#### LLOQ, ULOQ and quality control samples

The lower limit of quantification (LLOQ) for FAV and NTZ was calculated from the equation (3.3*SD/slope) and found to be concentrations of 4 μg mL^−1^ and 2 μg mL^−1^, and the concentrations of 60 μg mL^−1^ was revealed to be the upper limit of quantification for both FAV and NTZ which calculated from the equation (10*SD/slope). The concentrations 8, 25, and 50 μg mL^−1^ were exploited as the QC samples.

#### Specificity, selectivity, accuracy, precision, extraction recovery and stability

The comparison of HPLC chromatograms from spiked-plasma samples with each of the examined drugs at their LLOQ and IS (Fig. 2) using specimens of blank plasma obtained from six healthful participants indicated no interference between the evaluated drugs and plasma matrix, confirming the selectivity of the suggested procedure.

The obtained values of intraday and interday precision and accuracy were shown in Table 1. The intraday precision (%RSD) range was 1.02–1.90%. Interday %RSD range was 0.61–1.09%. The estimated recovery was also satisfactory. All of these findings disclosed reasonable accuracy and precision of the proposed method [67]. Besides, the calculated extraction recoveries were 100.14% (for FAV) and 98.87% (for NTZ) with acceptable %RSD values (Supplementary Table 1). In accordance with the stability results in Table 2, the determined concentrations were consistent under the aforementioned circumstances.

**Table 1:** Intra-day and inter-day precision and accuracy of FAV and NTZ in spiked human plasma

Concentration* (µg mL^−1^)	Intraday precision						Interday precision					
	FAV			NTZ			FAV			NTZ		
	Recovery %*	RSD%	Bias %**	Recovery %*	RSD%	Bias %**	Recovery %*	RSD%	Bias %**	Recovery %*	RSD%	Bias %**
8	103.78	1.40	3.78	102.35	1.90	2.35	102.42	1.03	2.42	102.02	0.85	2.02
25	101.78	1.31	1.78	101.72	1.69	1.72	101.47	0.61	1.47	101.24	1.09	1.24
50	102.97	1.26	2.97	102.74	1.02	2.74	101.73	0.99	1.73	101.46	0.72	1.46

^*^Average of 5 experiments

^**^Bias = [(measured concentration- true concentration)/ true concentration] × 100

**Table 2:** Stability results of FAV and NTZ in spiked human plasma at different conditions

Analyte	Concentration (µg mL^−1^	%Recovery		
		Bench top (6 h)	Autosampler for 24 h	Three freeze thaw cycle
FAV	8	106.15	108.33	108.87
	25	104.81	106.38	106.55
	50	103.00	104.70	104.75
Mean ± RSD		104.65 ± 1.85	106.47 ± 1.81	106.72 ± 2.07
NTZ	8	103.88	104.32	102.76
	25	101.48	105.40	102.73
	50	100.99	101.88	104.55
Mean ± RSD		102.11 ± 1.55	103.87 ± 1.81	103.35 ± 1.04

^*^Average of 5 determinations

#### Robustness

Robustness refers to a method's capacity to endure minor variations in chromatographic parameters such as changing percent of ethanol in the mobile phase (± 1%), alteration of formic acid concentration (± 0.01%) or modifying the flow rate (± 0.05 mL min^−1^). The impact of these modifications was calculated as the %RSD of the t_R_ value. Changes in the investigated parameters had no discernible impact on the t_R_ values of the resolved peaks (Supplementary Table 2).

#### System suitability testing parameters

The measured system suitability parameters of the developed HPLC method embracing resolution, tailing factor, and selectivity were acceptable as exhibited in Table 3 [68].

**Table 3:** System suitability testing parameters of the proposed HPLC methods for simultaneous determination of FAV and NTZ in spiked human plasma

Parameters	FAV	IS	NTZ	Reference value
Asymmetry factor	1.09	1.13	1.20	< 2
Selectivity (α)	1.54		1.72	α > 1
Resolution (R)	1.07		1.98	R > 1.5
Capacity factor	1.15	1.73	2.97	1–10
Column efficiency (N)	1430.01	1650.87	1890.77	Increasing with efficiency of separation
HETP	0.0174	0.0151	0.0132	The smaller the value, the higher the column efficiency

### Greenness assessment

Analytical performance typically focuses on criteria such as accuracy, precision, selectivity, sensitivity, and stability. Conversely, factors that deal with the environmental impact and worker safety are not usually taken into consideration. In the last decades, various green metrics were developed and applied for greenness evaluation. However, neither of the introduced metrics offered a complete greenness assessment for the entire of the analytical procedures.

Between the greenness assessment metrics, the NEMI tool provides better visualization and an unproblematic comparison of the results [29]. The NEMI technique evaluates a procedure based on four primary features comprise the toxicity, corrosivity of chemicals and reagents, associated dangers, and the volume of waste produced. A 4-quadrant pictogram is derived by referencing every feature. The developed HPLC method was succeeded to fulfill all quadrants of the NEMI pictogram by using of non-PBT, non-hazardous, noncorrosive (pH > 2) solvents and producing of waste less than 50 g, as demonstrated in Table 4.

**Table 4:** Greenness assessment of the proposed HPLC method for the determination of FAV and NTZ by Analytical Eco-scale, NEMI GAPI and AGREE approaches

Analytical eco-scale		Penalty points	NEMI pictogram	AGREE	ComplexGAPI
Reagents	Ethanol Consumed volume/sample = 4.4. mL Subtotal PP = 1 [solvent 10–100 mL] Signal word = 2 [Danger] No. of pictograms = 2	4			
	0.1% formic acid Consumed volume/sample = 0.0037 mL Subtotal PP = 1 [solvent < 10 mL] Signal word = 2 Danger No. of pictograms = 3	6			
Instruments	HPLC device (≤ 1.5 kWh per sample)	1			
	Column heating	2			
	Occupational hazard	0			
	Sonictor	1			
	Waste (< 1 mL)	3			
Total Penalty Points		17			
Eco-scale		83			

The Analytical Eco Scale is a tool utilized for environmental evaluation in analytical chemistry. It assesses the environmental impact of analytical procedures according to different criteria concerning sustainability, resource efficiency, and ecological compatibility. The estimated penalty points are subtracted from an initial value of 100 for determining the analytical Eco-scale metric [69]. The penalty points are calculated for each reagent based on its used mount and its hazardous and toxic properties. Additionally, penalty points are also given for the analyst's potential work environment, the quantity of energy utilized by instruments and the waste produced [70]. The Eco-scale results of the developed method (85) confirmed its excellent green environmental impact (Table 4).

Recently, a new downloadable greenness assessment software, AGREE was developed in accordance with GAC's twelve principles and guidelines [71]. In AGREE [31], automatic pictograms were generated that are organized into twelve parts, with the adjustable section width based on its importance. The overall score (ranging from 0 to 1) is displayed in in the circular pictogram's center. 

Table 4 shows the AGREE pictogram and its final score (0.68) assured a green impact of the developed method.

ComplexGAPI is a newer software utilizes extra health and safety considerations to offer a more thorough assessment for every phase of the quantitative analytical method [30, 32] including the preanalytical procedures. As there are extraction procedures and sample preparation steps must be followed before application of the proposed method, using the ComplexGAPI approach was necessary and provide a better evaluation than the other green chemistry metrics. As the developed method does not include synthetic procedures, so the product yield, purity and *E-factor* were not included. In ComplexGAPI assessment, a particular symbol utilizes five pentagrams and one hexagram ordered For the low, medium, and high environmental consequences, the colour scale goes from green to yellow to red. According to Table 4's interpretation of the ComplexGAPI pictogram for the constructed approach, the method has a beneficial effect on the environment because (10) fields were colored green, (9) yellow, and only (3) red. Additionally, it was clear that the developed method is green for pre- and during analytical steps.

The application of the four-assessment metrics: NEMI, Analytical Eco-Scale, AGREE, and ComplexGAPI, provides a comprehensive overview of the environmental sustainability of the created method. The consistent agreement among the four metrics proves the proposed method is environmentally sustainable.

## Conclusion

For the initial time, a straightforward, dependable, and accurate HPLC approach was established for the concurrent determination of the co-administered FAV and NTZ combination used in the treatment of COVID-19. The proposed method has many advantages, including that it is highly selective, sensitive, and reproducible. Furthermore, it can be used in daily clinical practice as well as pharmacokinetics and bioequivalence studies of the studied drugs. Likewise, the proposed method was validated in accordance with US-FDA guidelines, and it generated good results within acceptable limits. Also, it embraced using of environmentally safe green solvents and procedures that demonstrated an excellent greenness profile as confirmed by NEMI, Analytical Eco-scale AGREE, and ComlexGAPI green chemistry metrics. Lastly, even though the developed analytical method is the first one to estimate both FAV and NTZ simultaneously. But an extensive pharmacokinetic study for the their simultaneous use or COVID-19 management is still required.

## Supplementary Information

Supplementary material 1

## Data Availability

The data that support the findings of this study are available from the corresponding author (Amal B Ahmed) upon reasonable request.

## Abbreviations

NTZ: Nitazoxanide
FAV: Favipiravir
HPTLC: High-performance thin layer chromatography
GAPI: Green analytical procedure index
NEMI: National environmental method index
ICH: International council for harmonization
LLOQ: Lower limit of quantification
ULOQ: The upper limit of quantification
SD: Standard deviations
