# Supplementary material for: Eco-sustainable chromatographic method for the determination of favipiravir and nitazoxanide for COVID-19: application to human plasma
Source: BMC Chem. 2025 Jan 9;19(1):11. doi: 10.1186/s13065-024-01364-3 (PMC11714856; doi:10.1186/s13065-024-01364-3)
Supplement: Supplementary file 1 — Supplementary material 1 [file 13065_2024_1364_MOESM1_ESM.docx]

Supplementary Table 1: Results of extraction recovery of the studied drugs in spiked human plasma.

| **Concentration* (**µg mL^-1^**)** | **%Recovery**** | |
| --- | --- | --- |
|  | **FAV** | **NTZ** |
| **8** | 102.88 | 103.42 |
| **25** | 101.14 | 101.32 |
| **50** | 100.87 | 101.30 |
| **Mean± % RSD** | 101.63 ± 1.09 | 102.01 ± 1.22 |

*Average of 5 experiments

** Bias = [measured concentration/ true concentration] x100

Supplementary Table 2: Robustness of the proposed UHPLC method for the simultaneous determination of FAV and NTZ in spiked human plasma.

| **NTZ (%RSD)** | **FAV (%RSD)** | **For UHPLC** |
| --- | --- | --- |
| 0.32 | 0.59 | Formic acid concentration (0.1 ± 0.05% |
| 1.05 | 1.66 | Ethanol (45 ± 1%) |
| 0.96 | 1.62 | Flow rate (0.8 ± 0.05 mL min^-1^) |
